# Diagnostic magnetic resonance imaging characteristics of congenital mesoblastic nephroma: a retrospective multi-center International Society of Pediatric Oncology-Renal Tumor Study Group (SIOP-RTSG) radiology panel study

**DOI:** 10.1007/s00247-024-05918-4

**Published:** 2024-04-13

**Authors:** Justine N. van der Beek, Jens-Peter Schenk, Carlo Morosi, Tom A. Watson, Ana Coma, Norbert Graf, Tanzina Chowdhury, Gema L. Ramírez-Villar, Filippo Spreafico, Nils Welter, Kristina Dzhuma, Harm van Tinteren, Ronald R. de Krijger, Marry M. van den Heuvel-Eibrink, Annemieke S. Littooij

**Affiliations:** 1 Department of Radiology and Nuclear Medicine, University Medical Center Utrecht/Wilhelmina Children’s Hospital, Utrecht University, Heidelberglaan 100, 3584 CX Utrecht, The Netherlands; 2 Princess Máxima Center for Pediatric Oncology, Utrecht, The Netherlands; 3 Clinic of Diagnostic and Interventional Radiology, Division of Pediatric Radiology, Heidelberg University Hospital, Heidelberg, Germany; 4 Department of Radiology, Fondazione IRCCS Istituto Nazionale dei Tumori, Milan, Italy; 5 Department of Paediatric Radiology, Great Ormond Street Hospital for Children NHS Foundation Trust, London, UK; 6 Department of Pediatric Radiology, Hospital Vall d’Hebron, Barcelona, Spain; 7 Department of Pediatric Oncology & Hematology, Saarland University Medical Center and Saarland University Faculty of Medicine, Homburg, Germany; 8 Department of Haematology and Oncology, Great Ormond Street Hospital for Children NHS Foundation Trust, London, UK; 9 Department of Paediatric Oncology, Hospital Universitario Virgen del Rocío, Seville, Spain; 10 Pediatric Oncology Unit, Department of Medical Oncology and Hematology, Fondazione IRCCS Istituto Nazionale dei Tumori, Milan, Italy; 11 Developmental Biology and Cancer Department, University College London Great Ormond Street Institute of Child Health, London, UK; 12 Department of Paediatric Urology, Great Ormond Street Hospital for Children NHS Foundation Trust, London, UK; 13 Department of Pathology, University Medical Center Utrecht, Utrecht, The Netherlands; 14 Division of Child Health, Wilhelmina Children’s Hospital, Utrecht University, Utrecht, The Netherlands

**Keywords:** Congenital mesoblastic nephroma, Kidney neoplasms, Magnetic resonance imaging, Pediatrics, Radiology, Wilms tumor

## Abstract

**Background:**

Congenital mesoblastic nephroma is the most common solid renal tumor in neonates. Therefore, patients <3 months of age are advised to undergo upfront nephrectomy, whereas invasive procedures at diagnosis in patients ≥3 months of age are discouraged by the International Society of Pediatric Oncology-Renal Tumor Study Group (SIOP-RTSG). Nevertheless, discriminating congenital mesoblastic nephroma, especially from the more common Wilms tumor, solely based on imaging remains difficult. Recently, magnetic resonance imaging (MRI) has become the preferred modality. Studies focusing on MRI characteristics of congenital mesoblastic nephroma are limited.

**Objective:**

This study aims to identify diagnostic MRI characteristics of congenital mesoblastic nephroma in the largest series of patients to date.

**Materials and methods:**

In this retrospective multicenter study, five SIOP-RTSG national review radiologists identified 52 diagnostic MRIs of histologically proven congenital mesoblastic nephromas. MRI was performed following SIOP-RTSG protocols, while radiologists assessed their national cases using a validated case report form.

**Results:**

Patients (24/52 classic, 11/52 cellular, and 15/52 mixed type congenital mesoblastic nephroma, 2/52 unknown) had a median age of 1 month (range 1 day–3 months). Classic type congenital mesoblastic nephroma appeared homogeneous with a lack of hemorrhage, necrosis and/or cysts, showing a concentric ring sign in 14 (58.3%) patients. Cellular and mixed type congenital mesoblastic nephroma appeared more heterogeneous and were larger (311.6 and 174.2 cm^3^, respectively, versus 41.0 cm^3^ for the classic type (*P*<0.001)). All cases were predominantly T2-weighted isointense and T1-weighted hypointense, and mean overall apparent diffusion coefficient values ranged from 1.05–1.10×10^−3^ mm^2^/s.

**Conclusion:**

This retrospective international collaborative study showed classic type congenital mesoblastic nephroma predominantly presented as a homogeneous T2-weighted isointense mass with a typical concentric ring sign, whereas the cellular type appeared more heterogeneous. Future studies may use identified MRI characteristic of congenital mesoblastic nephroma for validation and for exploring the discriminative non-invasive value of MRI, especially from Wilms tumor.

**Graphical Abstract:**

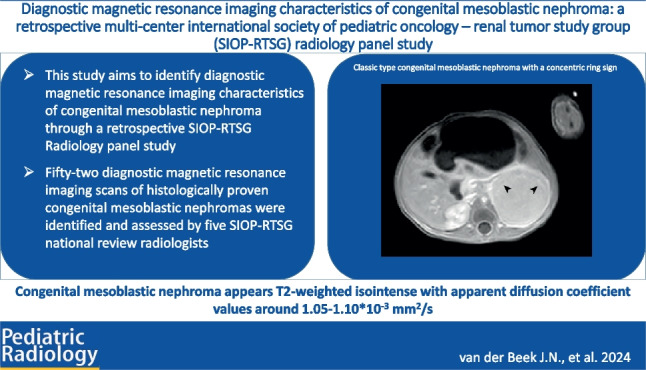

**Supplementary Information:**

The online version contains supplementary material available at 10.1007/s00247-024-05918-4.

## Introduction

Congenital mesoblastic nephroma accounts for only 2–5% of all pediatric renal tumors, whereas it is the most common solid renal tumor in neonates [1–5]. Approximately 75% of all cases are diagnosed in the first 6 months of life [5]. Congenital mesoblastic nephroma is considered to arise perinatally; however, only 11–15% have been reported to be detected through prenatal imaging [2, 6–8]. Congenital mesoblastic nephroma as a separate entity was first described by Bolande et al. and classified as a benign renal tumor [9, 10]. Nevertheless, given limited reports of metastatic behavior, it is currently best classified as a mesenchymal tumor with low malignant potential [2, 5, 11–14]. Morbidity and mortality are predominantly caused by associated polyhydramnios and other related paraneoplastic syndromes at presentation, whereas the tumor rarely metastasizes, mainly to the lungs, brain, liver, or bone [11, 15–20]. Histologically, three subtypes have been described [9]. The classic type consists of bland spindle cells, with few mitoses and no apparent hemorrhage and necrosis [2, 7, 21–23]. In contrast, the cellular type shows a high mitotic index and invasive growth pattern with areas of hemorrhage and necrosis [2, 3, 7, 17, 22]. In general, if both histological patterns are present, the lesion is classified as mixed type [2, 23, 24].

In children older than 6 months of age who have a renal tumor, preoperative chemotherapy for Wilms tumor is standard of care within the current Renal Tumor Study Group of the International Society of Pediatric Oncology (SIOP-RTSG) 2016 UMBRELLA protocol. In general, children <3 months of age undergo an upfront nephrectomy, which is the standard treatment for congenital mesoblastic nephroma [11, 25–27]. Wilms tumor is the most frequent pediatric renal tumor in the first decade of life, whereas neonatal cases are rare [28, 29]. Rhabdoid tumor of the kidney and clear cell sarcoma of the kidney are also predominantly diagnosed in the first years of life [2, 29–31]. As pediatric renal tumors preferably require different treatment approaches, and biopsies are not advocated by the SIOP-RTSG in young children, non-invasive discrimination through imaging is important in the diagnostic process, especially in infants >3 months of age [11, 29]. While prenatal and antenatal ultrasound (US) are the first-line modalities for diagnosis, magnetic resonance imaging (MRI) plays an increasingly important role due to the excellent soft-tissue contrast without use of ionizing radiation, and is therefore advocated as preferred modality within the SIOP-RTSG [6, 11, 28, 32].

Based on previous case reports and small retrospective studies, congenital mesoblastic nephroma is typically described as homogeneous and isointense to normal renal parenchyma [4, 7, 12, 14, 33]. The cellular type seems to be more heterogeneous, making differentiation from malignant tumors more difficult [3, 6, 8, 21, 28, 33–37]. Therefore, identification of specific MRI characteristics of congenital mesoblastic nephroma at diagnosis, especially in young children, is important for the discrimination from Wilms tumors and more aggressive non-Wilms tumor [11, 29, 34, 38]. This study aims to identify MRI characteristics that may be specific for congenital mesoblastic nephroma at diagnosis through a retrospective international multicenter SIOP-RTSG cohort study in the largest number of described patients to date.

## Materials and methods

### Patients

For this retrospective international multicenter study, five SIOP-RTSG national review radiologists (J.P.S. with 20 years of experience, A.S.L. with 15 years of experience, C.M. with 20 years of experience, T.A.W. with 11 years of experience, and A.C. with 10 years of experience) searched their center-specific and/or national databases for pediatric patients (0–17 years), diagnosed with a histologically proven congenital mesoblastic nephroma, and registered in SIOP (2001 or 2016 UMBRELLA or UK-IMPORT (United Kingdom-Improving Population Outcomes for Renal Tumors of childhood)) studies. Cases were included based on availability of a diagnostic MRI scan, which was performed as standard of care following SIOP-RTSG protocols, and histopathological assessment by national SIOP-RTSG pathologists following the afore mentioned active SIOP-RTSG protocols [39–42].

### Magnetic resonance imaging acquisition

Children were scanned in their local centers in Germany, The Netherlands, Italy, the UK, and Spain. Scan protocols were based on MRI guidelines as defined in SIOP-RTSG protocols, performed on predominantly 1.5-tesla (T) systems *(*Table 1) [29, 43]. Nine patients were scanned on 3T systems (*n*=7 in Germany, *n*=1 in the UK and *n*=1 in Spain). Imaging was predominantly performed on MRI scanners from Philips Medical Systems (Best, The Netherlands) and Siemens Healthineers (Erlangen, Germany); however, patient-specific information concerning manufacturers was not available for all cases. Diffusion weighted imaging (DWI), when available, was performed with a variety of *b*-values, with assessable apparent diffusion coefficient (ADC) maps. ADC values were obtained from one to four freehand drawn regions of interest (ROIs) in solid parts of the tumor.

**Table 1:** Average scan parameters per country

Parameters	T1W pre-/post-contrast	T2W	DWI
Germany (*n*=31 patients)			
Pulse sequence	TSE / GRE-VIBE	MVXD / TSE	ep
Slice orientation	Transversal	Transversal	Transversal / Coronal
Repetition time (ms)	6.8	2647.5	5800.0
Echo time (ms)	2.7	94.4	73.7
Slice thickness (mm)	3.0	3.5	4.0
Echo train length	1.0	27.5	34.5
Acquisition matrix	256\0\0\154^a^	256\0\0\182^a^	184\0\0\130^a^
*b*-values	*NA*	*NA*	0-50-400-800-1000^b^
The Netherlands (*n*=8 patients)			
Pulse sequence	GRE	fs MV / TSE	ep
Slice orientation	Transversal	Transversal / Coronal	Transversal
Repetition time (ms)	5.5	1319.1	2206.7
Echo time (ms)	2.7	100.0	72.7
Slice thickness (mm)	3.0	3.5	5.0
Echo train length	60.0	61.5	35.0
Acquisition matrix	232\0\0\233^a^	300\0\0\78^a^	88\0\0\70^a^
* b*-values	*NA*	*NA*	0-100-1000, 50-800 or 0-25-50-100-150-200-250-500-800-1000
Italy (*n*=5 patients)			
Pulse sequence	SE / GRE	SE	ep
Slice orientation	Transversal	Transversal	Transversal
Repetition time (ms)	5.3	3501.1	4749.0
Echo time (ms)	1.6	80.0	80.0
Slice thickness (mm)	3.0	3.5	4.0
Echo train length	8.0	34.0	35.0
Acquisition matrix	256\0\0\118^a^	256\0\0\256^a^	128\0\0\128
* b*-values	*NA*	*NA*	0-400-800-1000^b^
United Kingdom (*n*=6 patients)			
Pulse sequence	TFE / GRE	TSE	ep
Slice orientation	Transversal	Transversal	Transversal
Repetition time (ms)	7.0	3101.1	3600.0
Echo time (ms)	1.8	107.1	72.7
Slice thickness (mm)	3.5	3.0	4.5
Echo train length	1.0	63.5	35.0
Acquisition matrix	320\0\0\200^a^	0\256\256\0^a^	128\0\0\128^a^
* b*-values	*NA*	*NA*	0-50-800-1000^b^
Spain (*n*=2 patients)			
Pulse sequence	GRE	(T)SE	ep
Slice orientation	Transversal	Transversal	Transversal
Repetition time (ms)	4.6	2382.5	4054.7
Echo time (ms)	2.2	93.5	67.4
Slice thickness (mm)	3.0	4.0	4.0
Echo train length	32.0	34.0	46.0
Acquisition matrix	224\0\0\224^a^	272\0\0\113^a^	76\0\0\76^a^
* b*-values	*NA*	*NA*	50–800 or 100-50-600

^a^Wide variety of acquisition matrixes

^b^Different combinations of reported *b*-values

*DWI* diffusion-weighted imaging, *ep* echoplanar, *fs* fat suppression, *GRE* gradient echo, *mm* millimeter, *ms* milliseconds, *MV* multivane, radial sampling method, *NA* not applicable, *SE* spin echo, *TFE* turbo field echo, *TSE* turbo spin echo, *T1W* T1-weighted imaging, *T2W* T2-weighted imaging, *VIBE* volumetric interpolated breath-hold examination

Depending on their ability to cooperate and according to local standard of care procedures, children were awake, sedated or under general anesthesia. Gadolinium (Gadovist; Bayer BV, Leverkusen, Germany in cases with available details) and hyoscine butylbromide (Buscopan; Sanofi, Paris, France in cases with available details) were administered according to center specific regulations, based on the current general recommendations of the SIOP-RTSG/UK-IMPORT protocols [43].

### Image analysis

Each of the five national review radiologists assessed their own national congenital mesoblastic nephroma cases through a case report form (CRF), which was validated through an extensive interrater agreement study (median observed agreement of 77.0% (range 57.3–94.5%) among five raters) in a prior publication, showing satisfactory results [44]. This way, all included cases could be assessed by the individual national radiologist, without obligation for two observers per patient and without need for regulatory data sharing agreements. The CRF consisted of MRI characteristics potentially seen on diagnostic MRI scans of pediatric renal tumors, supported by an instruction file (Supplementary Material 1, Supplementary Material 2). Anonymization of CRFs was performed by the national radiologists through specific identification codes for each country, after which the CRFs were shared with the lead investigator, without the anonymization key. Although the focus of this study was descriptive, some patient and tumor characteristics were tested by congenital mesoblastic nephroma subtype. Because of the small numbers, non-parametric tests were applied (Fisher’s exact test and Kruskal-Wallis test). The results should be considered explorative, as no formal hypotheses were pre-specified. Results were considered significant at a *P*-value <0.05. Statistical analyses were performed in SPSS (version 27.0, IBM, NY, US).

## Results

### Patient characteristics

Fifty-two patients were included, originating from Germany (*n*=31), Italy (*n*=5), Spain (*n*=2), The Netherlands (*n*=8), and the UK (*n*=6) (Table 2). Twenty-four (46.2%) patients were diagnosed with classic type congenital mesoblastic nephroma, 11 (21.2%) patients with cellular type congenital mesoblastic nephroma, and 15 (28.8%) patients with mixed type congenital mesoblastic nephroma. The histological subtype was unknown for two patients. The median age of all patients (*n*=52) was 1 month (range 1 day–23 months), whereas the highest median age was seen in the cellular type (2 months, range 4 days–23 months) (Table 2). Twelve of the 52 (23%) patients were older than 3 months at time of diagnosis, including 11 patients with cellular and mixed type congenital mesoblastic nephroma. In total, 30/52 (57.7%) patients were male (Table 2). There was no suspicion of positive regional lymph nodes in any of the patients; however, one patient with cellular congenital mesoblastic nephroma showed peri-aortic supra-diaphragmatic lymph nodes, suspicious for metastatic disease, but not histologically confirmed.

**Table 2:** Magnetic resonance imaging characteristics of the included pediatric patients (*n*=52)^a^ with congenital mesoblastic nephroma, reported for classic, cellular, and mixed type

Characteristics		Classic (*n*=24)	Cellular (*n*=11)	Mixed (*n*=15)
Origin of included patients	Germany	14	6	11
	The Netherlands	3	3	2
	Italy	4	0	1
	United Kingdom	2	2	0
	Spain	1	0	1
Clinical characteristics	Median age in months *(range)*^*b*^	0 *(1d-6 months)*	2 (*4d-23 months)*	1 *(2d-9 months)*
	Sex *(male)*	13 (54.2%)	6 (54.5%)	9 (60.0%)
	Tumor side *(right)*	11 (45.8%)	6 (54.5%)	7 (46.7%)
	Metastatic disease	0 (0%)	1 (9.1%)^c^	0 (0%)
General tumor characteristics on MRI	Median tumor volume *(cm*^*3*^*, range)*^d^	41.0 (1.7-156.0)	311.6 (32.7-998.6)	174.2 (22.8-876.1)
	Location of the tumor			
	· Central	10 (41.7%)	5 (45.5%)	8 (53.4%)
	· Peripheral	14 (58.3%)	5 (45.5%)	5 (33.3%)
	· Indistinguishable	0 (0%)	1 (9.0%)	2 (13.3%)
Growth pattern on MRI	Tumor margins *(well-/ill-defined)*	12 (50.0%) /	5 (45.5%) /	10 (66.7%) /
		12 (50.0%)	6 (54.5%)	5 (33.3%)
	(Pseudo)capsule	5 (20.8%)	1 (9.1%)	3 (20.0%)
	Breach of the tumor capsule	1 (4.2%)	4 (36.4%)	4 (26.7%)
	Intra-peritoneal spread	0 (0%)	0 (0%)	1 (6.7%)
	Infiltrative growth pattern	3 (12.5%)	3 (27.3%)	4 (26.7%)
Solid tumor characteristics on MRI	T2-weighted pattern^d^			
	· Homogeneous	21 (87.5%)	2 (20.0%)^e^	5 (33.3%)
	· Heterogeneous	3 (12.5%)	8 (80.0%)	10 (66.7%)
	T2-weighted intensity			
	· Hyperintense	7 (29.2%)	5 (50.0%)^e^	5 (33.3%)
	· Hypointense	0 (0%)	0 (%)	1 (6.7%)
	· Isointense	17 (70.8%)	5 (50.0%)	9 (60.0%)
	T1-weighted pattern^d^			
	· Homogeneous	23 (95.8%)	3 (27.3%)	9 (60.0%)
	· Heterogeneous	1 (4.2%)	8 (72.7%)	6 (40.0%)
	T1-weighted intensity			
	· Hyperintense	0 (0.0%)	0 (0.0%)	0 (0.0%)
	· Hypointense	16 (66.7%)	8 (72.7%)	12 (80.0%)
	· Isointense	8 (33.3%)	3 (27.3%)	3 (20.0%)
	Hemorrhage / Necrosis^d^	0 (%)	8 (72.7%)	5 (33.3%)
	· Limited	*NA*	· 4 (50.0%)	· 5 (100%)
	· More extensive	*NA*	· 4 (50.0%)	· 0 (%)
	Cysts	0 (0.0%)	8 (72.7%)	5 (33.3%)
	Subcapsular fluid	1 (4.2%)	2 (18.2%)	1 (6.7%)
	Increased vascularity	3 (12.5%)	2 (18.2%)	3 (20.0%)
	Concentric ring sign	14 (58.3%)	1 (9.1%)	5 (33.3%)
	Enhancement pattern^d^			
	· Homogeneous	17 (70.8%)	3 (27.3%)	6 (40.0%)
	· Heterogeneous	3 (12.5%)	6 (54.5%)	7 (46.7%)
	· No contrast-enhanced imaging	4 (16.7%)	2 (18.2%)	2 (13.3%)
DWI	Overall mean ADC-value (*10^*-3*^ *mm*^*2*^*/s, range*)	(*n*=17) 1.05 (0.74–1.36)	(*n*=9) 1.10 (0.77–1.62)	(*n*=11) 1.05 (0.88–1.41)

^a^The histological subtype of two patients was unknown, resulting in the analysis of 50 patients for general magnetic resonance imaging characteristics, and 37 patients for apparent diffusion coefficient values on diffusion-weighted imaging, ^b^Twelve patients had an age >3 months, with only two children with cellular type congenital mesoblastic nephroma aged > 1 year (18 months and 23 months), ^c^The patient showed peri-aortal supra-diaphragmatic lymph-nodes, suspected of metastases, but not histologically confirmed, ^d^Statistically significant (*P* < 0.05) concerning differentiation between classic-, cellular- and mixed type congenital mesoblastic nephroma following the Kruskal-Wallis rank sum test and Fisher’s exact test, ^e^In one patient with cellular type congenital mesoblastic nephroma T2-weighted imaging was not performed

*ADC* apparent diffusion coefficient, *d* days, *DWI* diffusion-weighted imaging, *MRI* magnetic resonance imaging, *NA* not applicable

### General tumor characteristics and growth pattern on magnetic resonance imaging

The median tumor volume was largest for the cellular type (311.6 cm^3^, range 32.7–998.6 cm^3^), and smallest for the mixed (174.2 cm^3^, range 22.8–876.1 cm^3^) and the classic types (41.0 cm^3^, range 1.7–156.0 cm^3^) (*P*<0.001) *(*Table 2). Location of the tumor was equally distributed over a central and peripheral location in the kidney (26/52 peripheral), with no evident differences for subtypes. Tumor margins were well-defined in 28/52 (53.8%) lesions, also evenly distributed among the subtypes. A pseudocapsule was observed in one patient with the cellular type (1/11, 9.1%), and more often in classic and mixed type (20.8% and 20.0%, respectively). An infiltrative growth pattern was observed in cellular type (3/11, 27.3%) and mixed type congenital mesoblastic nephroma (4/15, 26.7%) (Table 2). There were no cases presenting with venous invasion.

### Solid tumor characteristics on magnetic resonance imaging

**Fig. 1:**
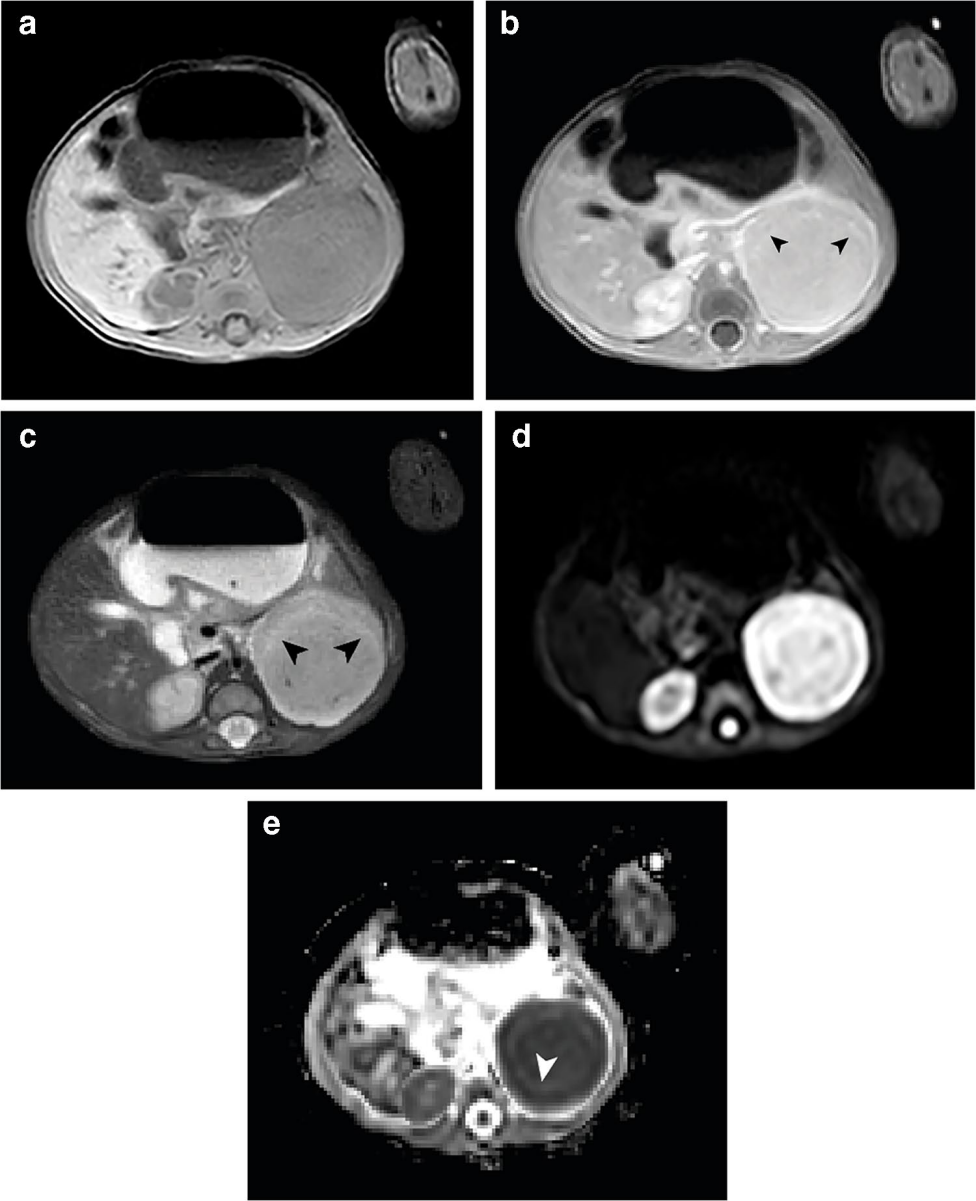
Magnetic resonance imaging of a 1-month-old boy with a left-sided classic type congenital mesoblastic nephroma with a volume of 44 cm^3^. **a **Axial T1-weighted image shows a homogeneous and isointense tumor. **b **Contrast-enhanced axial T1-weighted image shows homogeneous enhancement, with the concentric ring sign (*arrowheads*). **c **This homogeneity of the tumor as well as the hyperintense ring sign are also seen on the axial T2-weighted sequence, with an isointense appearance (*arrowheads*). **d **On the axial diffusion-weighted imaging b1000 sequence the tumor shows a homogeneous high intensity. **e **On the calculated axial apparent diffusion coefficient map, the tumor shows no evident increased diffusion restriction compared to the healthy contralateral renal tissue, with a median apparent diffusion coefficient value of solid parts of the tumor of 1.100×10^−3^ mm^2^/s. A hyperintense concentric ring is observed (*arrowhead*)

**Fig. 2:**
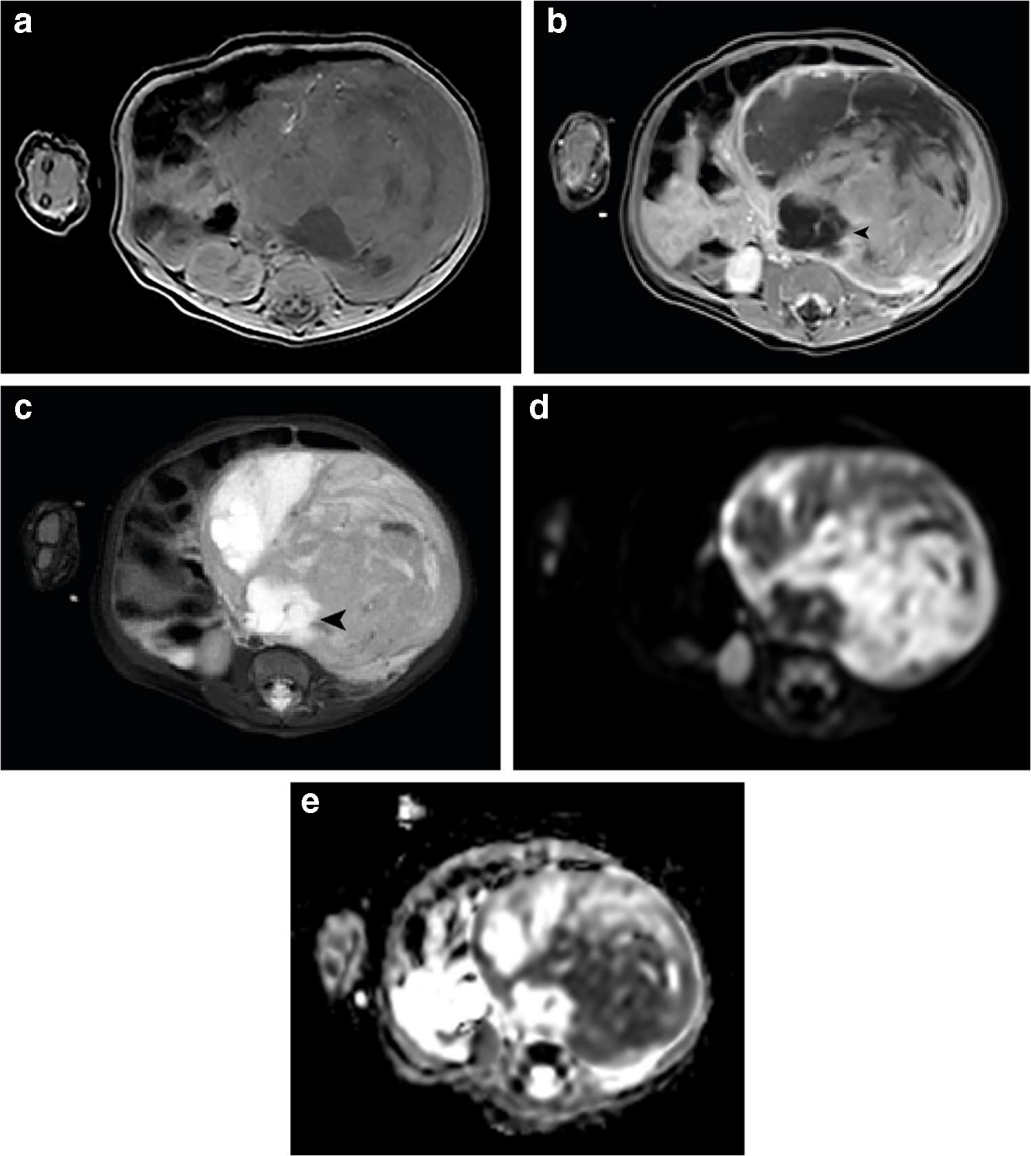
Magnetic resonance imaging of a 2-month-old girl with a left-sided cellular type congenital mesoblastic nephroma with a volume of 312 cm^3^. **a **Axial T1-weighted image shows a heterogeneous and isointense tumor. **b **Contrast-enhanced axial T1-weighted image shows heterogeneous enhancement, with some large cysts (*arrowhead*). **c **Axial T2-weighted image shows evident hemorrhage/necrosis and again isointensity of the solid components of the tumor, with hyperintense cysts (*arrowhead*). **d **On the axial diffusion weighted imaging b1000 sequence the tumor appears heterogeneous with varying diffusion restriction. **e **On the axial calculated apparent diffusion coefficient map, the solid parts of the lesion show considerable restricted diffusion compared to the (healthy) renal tissue, with a median apparent diffusion coefficient value of 1.000×10^−3^ mm^2^/s

On T2-weighted (T2W) imaging, all subtypes showed a predominant isointense appearance compared to the renal parenchyma, whereas T2W hypointensity was limited to only one case of mixed type congenital mesoblastic nephroma. On T1-weighted (T1W) imaging, none of the tumors appeared hyperintense, whereas most subtypes showed T1-weighted hypointensity (66.7% for classic type, 72.7% for cellular type and 80.0% for mixed type) (Table 2; Figs. 1 and 2). On all sequences, the classic type appeared predominantly homogeneous (70.8% on T1W contrast-enhanced imaging, *P*<0.001), whereas the cellular type and mixed type appeared predominantly heterogeneous after contrast enhancement (54.5% and 46.7%, respectively) (*P*=0.010) *(*Table 2; Figs. 1 and 2). Similarly, the number of cases with hemorrhage and/or necrosis (8/11, 72.7%) and cysts (8/11, 72.7%) was highest in the cellular type, whereas these imaging characteristics were absent in all classic type cases (*P*<0.001) (Figs. 1 and 2). The concentric ring sign was seen in 14/24 classic type cases (58.3%) and was less common in the other subtypes (Table 2; Fig. 1). None of the tumors showed fatty tissue.

### Diffusion weighted imaging

In 39/52 (75.0%) cases, DWI was available for measurement of ADC values based on ROIs of solid parts of the tumor. The overall mean ADC-value for classic type congenital mesoblastic nephroma was 1.05×10^−3^ mm^2^/s (range 0.74–1.36×10^−3^ mm^2^/s) (Fig. 1). For the cellular type, overall ADC-values ranged from 0.77–1.62×10^−3^ (overall mean 1.10×10^−3^ mm^2^/s), and mixed type cases showed an overall mean of 1.05×10^−3^ mm^2^/s (range 0.88–1.41×10^−3^ mm^2^/s) (Table 2; Fig. 2). In two patients with unknown subtype, the mean overall ADC-values were 0.74×10^−3^ mm^2^/s and 1.62×10^−3^ mm^2^/s.

## Discussion

Given the evidence-based SIOP-RTSG guidelines advocating against invasive procedures to determine histology at diagnosis in the majority of children with renal tumors, imaging plays an increasingly important role in the non-invasive discrimination of pediatric renal tumors [8, 25, 34]. This retrospective multicenter study illustrates the MRI characteristics of congenital mesoblastic nephroma in an international cohort, focusing on its different subtypes and identifying potentially specific MRI characteristics of this rare neonatal pediatric renal tumor. Although we know congenital mesoblastic nephroma accounts for the majority of prenatal and neonatal renal tumors, and outcome is excellent with reported outcome rates of 95–100%, early recognition and discrimination from more malignant pediatric renal tumors is important, especially in children >3 months of age [1, 3, 11, 15–17, 29]. T2W isointensity particularly, appears to be potentially discriminating in the differentiation of congenital mesoblastic nephroma from the often T2W hyperintense Wilms tumor. Nonetheless, while the classic type congenital mesoblastic nephroma often appears homogeneous on imaging, the cellular and mixed type show more overlapping imaging MRI characteristics with malignant pediatric renal tumors in the same age range, such as Wilms tumor, rhabdoid tumor of the kidney, and clear cell sarcoma of the kidney [2, 3, 6, 8, 11, 21, 31, 37, 44].

Above the age of 3 months, a combination of certain clinical, radiological, and biochemical criteria are usually used to decide if a diagnostic cutting needle biopsy is indicated [26, 27]. In general, some studies have indicated the incidence of congenital mesoblastic nephroma is higher in males than females, whereas we only saw a slight predominance of male patients in our study (57.7%) [33, 45–47]. Metastases are described in approximately 2% of patients with the cellular type, which is in line with only one suspected metastatic case in this study [11, 13, 15, 18, 21, 35, 37]. Although its malignant potential remains a topic of debate, the rarity of metastases may be a discriminating factor between congenital mesoblastic nephroma and malignant renal tumors, especially in case of the classic type [5, 6, 8, 11, 14, 48]. Nonetheless, a total nephrectomy is the indicated treatment for all subtypes, especially given the increased likelihood of local recurrence in approximately 5% of the patients after a partial nephrectomy due to positive margins [11, 13, 15, 18].

We found that the classic type congenital mesoblastic nephroma often appears as a solid, well-defined, and homogeneous tumor, showing isointensity compared to the renal parenchyma on T2W imaging. The homogeneous appearance is predominantly caused by a lack of hemorrhage, necrotic, and/or cystic changes, which is in line with previous studies [23, 28, 34]. The concentric ring sign, also known as “double layer sign,” was present in more than half of our classic type cases. This recognizable ring pattern, appearing hypoechoic on the abdominal ultrasound, in contrast to the isoechoic tumor tissue, has been predominantly mentioned in the context of the classic variant, and is hypothesized to be caused by compressed kidney tissue and dilated blood vessels [49–51]. Nevertheless, it does not appear to be specific to classic type congenital mesoblastic nephroma, as we also reported the characteristic in cellular and mixed cases in this study, in line with Daniel et al. [3, 52].

In case reports and small series, the cellular type congenital mesoblastic nephroma is suggested to be more heterogeneous on MRI, due to cystic, hemorrhagic, and/or necrotic components, in general showing a more aggressive growth pattern compared to the classic variant, as well as a larger size [3, 21, 23, 33, 36, 48]. Our study confirmed these findings, again stressing the difficulty to discriminate this variant from the often large, heterogeneous Wilms tumour [6, 11, 13, 21, 28, 48]. Also, this cohort of patients with cellular and mixed type congenital mesoblastic nephroma seemed to be older, indicating an overlap in age with the malignant Wilms and non-Wilms tumors [31, 34]. Nevertheless, Wilms tumors are often described as T2W hyperintense, which might indicate T2W isointensity as slightly discriminative for congenital mesoblastic nephroma in general [21, 22, 24, 53]. Rhabdoid tumors of the kidney are reported to be small, mainly T2W hypointense and often show infiltrative and aggressive features, potentially discriminating them from congenital mesoblastic nephroma as well as from Wilms tumors [44, 54]. Finally, no additional potentially discriminative MRI characteristics were identified for mixed type congenital mesoblastic nephroma, concerning both differentiation from the classic and cellular type and from other renal tumor types.

Whereas solid tumor characteristics and T1W and T2W imaging are predominantly used to identify abnormal and potentially specific characteristics of tumors, DWI and ADC values might contribute through the semi-quantification of cellularity [6, 34, 50]. Nevertheless, very limited studies have reported ADC-values of congenital mesoblastic nephroma, and conclusions are limited to the presence or absence of diffusion restriction in general [6, 48]. We showed ADC values for all subtypes to be approximately the same, with overall means ranging from 1.05 to 1.10×10^−3^ mm^2^/s, indicating moderate diffusion restriction in most tumors. ADC values around this range have also been described for clear cell sarcoma of the kidney, as well as stromal type Wilms tumor, whereas in general, more aggressive Wilms tumor subtypes show a higher diffusion restriction [44, 55, 56].

Our study has several limitations, predominantly related to its international setting and retrospective nature. While this could lead to potential information bias and variability, there was excellent interrater agreement among the included radiologists, who are experts in the field of imaging of pediatric renal tumors [44]. Also, the design of this study and related inclusion in different SIOP-RTSG protocols over the past decades affects the extent of national registration and center-specific choice of cross-sectional imaging modalities, which may have led to registration bias, potentially resulting in a disproportionate number of included patients per country, nonetheless also taking into consideration differences in population. Furthermore, in international studies, variability on the level of the patient and the MRI, reflected in the heterogeneity of reported scan parameters, has to be taken into consideration [57]. While this might not influence the reported results, it limits the possibility for statistical analysis in DWI data, given non-comparable ADC maps based on a variety of *b*-values [58]. Concerning clinical characteristics of the patients, this study was not designed to report on prenatal imaging or outcome, therefore lacking information on prenatal diagnoses and survival of the included patients. Also, despite the international setting of this study, numbers remained low and distribution of subtypes may not be in line with percentages reported by for instance a review by Gooskens et al., who showed a higher percentage of cellular type congenital mesoblastic nephroma (42%) [2]. Finally, statistical analysis of the discriminative value of MRI characteristics in the differentiation of subtypes of congenital mesoblastic nephroma was limited due to these relatively low numbers, while the differentiation from other pediatric renal tumors could not be analyzed based on the design of this predominantly descriptive study.

## Conclusion

Early non-invasive diagnosis of congenital mesoblastic nephroma based on MRI, as well as increasing knowledge of potential indications for a cutting needle biopsy, could be beneficial for increasing outcome and reducing treatment-related toxicity in pediatric renal tumor patients. This study forms the basis for future studies which may focus on the validation of identified potentially specific MRI characteristics in the light of differentiating congenital mesoblastic nephroma from Wilms tumors and other non-Wilms tumors, further exploring the discriminative value of MRI in pediatric renal tumor patients (Table 3).

**Table 3:** Overview of identified potentially specific magnetic resonance imaging and diffusion-weighted imaging characteristics of congenital mesoblastic nephroma in light of the differentiation of subtypes and the differentiation from Wilms tumor

Tumor type	Identified potentially specific MRI- and DWI-characteristics of congenital mesoblastic nephroma subtypes	Most common MRI- and DWI- characteristics of Wilms tumor^a^
Classic type	• Age predominantly <3 months^b^ • Concentric ring sign • Small tumor volume • T2W isointensity • Homogeneous enhancement • ADC-values around 1.05–1.10*10^-3^ mm^2^/s • Absence of metastases • Lack of (pseudo)capsule • Lack of venous invasion / tumor thrombus	• (Pseudo)capsule • Large, solid tumor • T1W hypointensity • T2W hyperintensity • Heterogeneous enhancement due to hemorrhagic/necrotic components • Varying ADC-values related to histopathology • Possible tumor thrombus • Pulmonary metastases • Bilateral disease
Cellular type	• Potentially older children (up to 2 years of age)^b^ • Large tumor volume • T2W isointensity • Heterogeneous enhancement due to potential presence of hemorrhage and/or necrosis • ADC-values around 1.05–1.10*10^-3^ mm^2^/s • Metastases are rare, but do occur • Lack of venous invasion / tumor thrombus	
Mixed type	• Characteristics similar to cellular type congenital mesoblastic nephroma	

^a^The magnetic resonance imaging characteristics reported in this table are the most common and general characteristics of Wilms tumor, but the great variety of potentially identifiable characteristics of Wilms tumors always needs to be taken into consideration; a non-specific presentation of a Wilms tumor remains more common than a specific presentation of a non-Wilms tumor; ^b^Age is not an MRI-characteristic, nevertheless, the higher range of age in cellular type congenital mesoblastic nephroma might be taken into consideration combined with the other identified potentially specific magnetic resonance imaging and diffusion weighted imaging characteristics

*ADC* apparent diffusion coefficient, *DWI* diffusion-weighted imaging, *MRI* magnetic resonance imaging, *T1W* T1-weighted, *T2W* T2-weighted

Since MRI is the preferred imaging modality for children with a renal tumor within the SIOP-RTSG, MRI characteristics of the different potential diagnoses need to be further explored. This study describes the MRI features of congenital mesoblastic nephroma at initial diagnosis through international collaboration of the SIOP-RTSG radiology panel in the largest retrospective series so far. Although age appears to remain the most important clinical characteristic to discriminate congenital mesoblastic nephroma from other pediatric renal tumor types, this study showed that homogeneity and a concentric ring sign quite specifically indicate a classic type. The cellular type can be discriminated from the classic variant based on its heterogeneity and larger size, whereas the T2W isointensity of all subtypes of congenital mesoblastic nephroma could be taken into consideration in the discrimination from more malignant pediatric renal tumors. Finally, the role of DWI and ADC values has been further explored, showing an almost equal ADC value for all congenital mesoblastic nephroma subtypes, which for example, could be used to discriminate from more aggressive pediatric renal tumors with high cellularity.

### Electronic supplementary material

Below is the link to the electronic supplementary material.

Supplementary Material 1

Supplementary Material 2

## Data Availability

The data supporting the findings of this study are available from the International Society of Pediatric Oncology – Renal Tumor Study Group (SIOP-RTSG) office following standard access procedures upon reasonable request.
