# Supplementary material for: Diagnostic magnetic resonance imaging characteristics of congenital mesoblastic nephroma: a retrospective multi-center International Society of Pediatric Oncology-Renal Tumor Study Group (SIOP-RTSG) radiology panel study
Source: Pediatr Radiol. 2024 Apr 13;54(6):965–76. doi: 10.1007/s00247-024-05918-4 (PMC11111520; doi:10.1007/s00247-024-05918-4)
Supplement: Supplementary file 1 — Supplementary Material 1 [file 247_2024_5918_MOESM1_ESM.pdf]

Case Report Form – Retrospective study
MRI-findings – Patient characteristics

STUDY NUMBER

.. R .. .. ..

Type of CMN	☐ Classic	☐ Cellular	☐ Mixed
	☐ Unknown		
Age at diagnosis *(months, rounded down)*	[ ]		
Sex	☐ Male	☐ Female	
Bilateral tumor at presentation	☐ Yes	☐ No	
• If yes: Study nr. contralateral tumor	… R … … …	☐ NA	
Tumor side	☐ Right	☐ Left	
Metastatic disease at diagnosis	☐ Yes	☐ No	☐ NA
• If metastatic disease: Site(s) of metastasis/metastases	[ ]		

Other remarks concerning the patient characteristics and diagnosis:

Case Report Form – Retrospective study
MRI-findings – Imaging characteristics at diagnosis (1)

STUDY NUMBER

.. R .. .. ..

Number of lesions on MRI at diagnosis ☐ 1 ☐ 2 ☐ More: [ ]

• Lesion nr. [ ]

Dimensions of the lesion *(three dimensions; cm, one decimal)* ………… * ………… * …………

Location of the lesion in the kidney ☐ Central ☐ Peripheral ☐ Indistinguishable / Impossible to determine

Regional lymph nodes (enlarged) ☐ Yes ☐ No ☐ NA

Additional information lymph nodes (size, enhancing, etc) [ ] ☐ NA

Margins ☐ Well-defined ☐ Ill-defined ☐ NA

(Pseudo) capsule ☐ Yes ☐ No ☐ NA

Growth pattern

Breach of the tumor capsule ☐ Yes ☐ No ☐ NA

Evidence of intra-peritoneal tumor spread ☐ Yes ☐ No ☐ NA

Infiltrative growth pattern ☐ Yes ☐ No ☐ NA

Venous invasion / Tumor thrombus ☐ Yes ☐ No ☐ NA

Tumor characteristics of <u>solid components</u>

T2-weighted imaging *(compared to renal cortex)*
- ☐ Homogeneous ☐ Heterogeneous ☐ Other: [ ]
- ☐ Hyper-intense ☐ Hypo-intense ☐ Iso-intense

T1-weighted imaging *(compared to renal cortex)*
- ☐ Homogeneous ☐ Heterogeneous ☐ Other: [ ]
- ☐ Hyper-intense ☐ Hypo-intense ☐ Iso-intense

Hemorrhage / Necrosis ☐ Yes ☐ No ☐ NA
- If yes: ☐ Limited ☐ More extensive

Cysts ☐ Yes ☐ No ☐ NA
- If yes: specification/size of the cysts: [ ] ☐ NA
- In case of a (completely) cystic tumor: Septation ☐ Yes ☐ No ☐ NA

Case Report Form – Retrospective study
MRI-findings – Imaging characteristics at diagnosis (2)

STUDY NUMBER

.. R .. .. ..

• Lesion nr. ☐

Tumor characteristics of <u>solid components</u> *(continuation)*

Fatty tissue	☐ Yes	☐ No	☐ NA
Subcapsular fluid	☐ Yes	☐ No	☐ NA
Increased vascularity	☐ Yes	☐ No	☐ NA
Concentric ring sign / Double-layer sign	☐ Yes	☐ No	☐ NA

Enhancement

Enhancement pattern
- ☐ Homogeneous
- ☐ Heterogeneous
- ☐ Band-like areas of late or non-enhancement
- ☐ No enhancement
- ☐ Other: ☐

Other remarks concerning the characteristics and appearance of the lesion(s):

Case Report Form – Retrospective study
MRI-findings – Imaging characteristics at diagnosis (3)

STUDY NUMBER

.. R .. .. ..

- Lesion nr. ☐

DWI-MRI available for review? ☐ Yes ☐ No

- If yes:

Diffusion restriction of the solid part of the lesion compared to healthy (contralateral) renal tissue ☐ Yes ☐ No

If available:

b-values used for ADC calculation *(lowest-highest)* b ☐ / b ☐

- ADC-value of first representative ROI with tumor tissue *($*10^{-3}$ mm^2/s, two decimals)* ☐

Dimensions of the first representative ROI ☐ Diameter *(cm)* ☐ or ☐ Surface *(cm^2)* ☐

- ADC-value of second representative ROI with tumor tissue *($*10^{-3}$ mm^2/s, two decimals)* ☐

Dimensions of the second representative ROI ☐ Diameter *(cm)* ☐ or ☐ Surface *(cm^2)* ☐

- ADC-value of third representative ROI with tumor tissue *($*10^{-3}$ mm^2/s, two decimals)* ☐

Dimensions of the third representative ROI ☐ Diameter *(cm)* ☐ or ☐ Surface *(cm^2)* ☐

- ADC-value of fourth representative ROI with tumor tissue *($*10^{-3}$ mm^2/s, two decimals)* ☐

Dimensions of the fourth representative ROI ☐ Diameter *(cm)* ☐ or ☐ Surface *(cm^2)* ☐
