# Supplementary material for: Diagnostic magnetic resonance imaging characteristics of congenital mesoblastic nephroma: a retrospective multi-center International Society of Pediatric Oncology-Renal Tumor Study Group (SIOP-RTSG) radiology panel study
Source: Pediatr Radiol. 2024 Apr 13;54(6):965–76. doi: 10.1007/s00247-024-05918-4 (PMC11111520; doi:10.1007/s00247-024-05918-4)
Supplement: Supplementary file 2 — Supplementary Material 2 [file 247_2024_5918_MOESM2_ESM.pdf]

Case Report Form Retrospective study – Instructions

Patient characteristics

General instructions and CRF page 1

Please use the following patient ID’s, and keep the anonymization key to yourself.

Country	Specific international study ID
Germany	GR001, GR002, GR003, etc.
United Kingdom	UR001, UR002, UR003, etc.
Spain	SR001, SR002, SR003, etc.
Italy	IR001, IR002, IR003, etc.
The Netherlands	NR001, NR002, NR003, etc.

If available, please indicate the type of CMN (classic, cellular or mixed) on the first page of the CRF. Indicate the age at diagnosis (in months) and sex of the patient. If you don’t have the age at diagnosis in months at your disposal, please try to indicate age at diagnosis in years (and describe the age is in years).

In case of a bilateral tumor, please consider these tumors as two separate cases with two separate CRFs and specify the study ID of the contralateral tumor.
If you have any other useful remarks concerning patient characteristics (i.e. treatment, highlights in the diagnostic process, etc.), please indicate them in the designated space

MRI-findings – Imaging characteristics at diagnosis

CRF page 2

Indicate the number of lesions on MRI at diagnosis. In case of multiple separate lesions of the tumor:
- The first lesion is the most cranial lesion, the last lesion is the most caudal lesion.
- Please complete page 2-4 separately for each lesion of the tumor. Please indicate the number of the lesion on each separate form. So in case of 2 lesions, complete page 2-4 with lesion nr. 1 indicated on each page, and complete page 2-4 with lesion nr. 2 indicated on each page.

General instructions:
- Indicate whether the characteristic was present on the MRI-scan.
- Use ‘other remarks concerning the characteristics of the tumor on MRI’ on page 3 for important details, additional information and anything of value for the characterization of the tumor type on MRI.
- In case the presence/absence of the characteristic cannot be determined, answer ‘Not Applicable’ (NA) and explain in ‘other remarks’ why the assessment of the characteristic was not possible.

Dimensions of the lesion are given in cm with one decimal.
Enlarged ***regional lymph nodes*** are identified based on their size (≥10 mm in the short axis).
A ***(pseudo)capsule*** shows a low signal intensity at T2W imaging.

Growth pattern
Breach of tumor capsule: Focal disruption of the capsule, with evidence of the tumor extending beyond the tumor capsule, but not being a tumor lobule. Discontinuation of the tumor 'capsule' does not automatically indicate extension of tumor into the peritoneum.
Evidence of intra-peritoneal spread of the tumor: Unequivocal spread of the tumor into the peritoneum, which can be seen by intra-peritoneal tumor depositions and/or large hematoperitoneum.
Infiltrative growth pattern: A poorly defined interface with the normal parenchyma and markedly irregular (non-elliptical/non-spherical) shape in one or more distinct/unequivocal areas.
Venous invasion / Tumor thrombus: Extension of the tumor into the renal vein and/or inferior vena cava.

Tumor characteristics of **solid components**
Presentation on different sequences:
- Answer each separate line (·) if multiple lines with answers are given after a question.
- A lesion can be classified as 'homogeneous' when ≥80% of the lesion has a homogeneous appearance on the MRI-sequence. Please specify the presentation of the lesion on the concerning sequence if the lesion cannot be described as homogeneous or heterogeneous.
- Hyper-intensity, hypo-intensity or iso-intensity should be determined comparing the lesion to the healthy (contralateral) renal cortex.

Hemorrhage / Necrosis: Hyper-intensity on non-contrast T1W imaging with a lack of enhancement on post-contrast imaging and variable (hypo-intense or hyper-intense) signal on T2W imaging.
If present/yes:
- Limited = Small and/or distinct foci of hemorrhage/necrosis
- More extensive = More than just foci of hemorrhage/necrosis

Cysts: Often hypo-intense on T1W imaging, hyper-intense on T2W imaging and non-enhancing on post-contrast imaging, except when cysts contain internal hemorrhage.
Septation: Septations can only be assessed in 'completely cystic tumors'. In case of this appearance, thin linear structures dividing different cysts or contained within individual cysts indicate the presence of septation.

CRF page 3

Tumor characteristics of solid components *(continuation)*
Fatty tissue: Using the sequences suitable, the lesion shows an indication/the presence of fatty tissue.
Subcapsular fluid: Fluid located subcapsular, showing the usual characteristics of fluid on the MRI.
Increased vascularity: An increased number of vessels, often with flow void on T2W imaging.

Concentric ring sign / Double-layer sign: A hyper- or hypo-intense 'ring' as a result of the vascular pattern of the CMN, showing a boundary/boundaries of enhancement. The sign is often seen as hypoechoic ring on abdominal US, but is also described on CT, and can be seen on sequences of MRI. Please find examples below.

Abdominal US

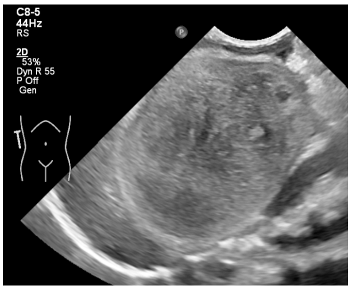

DWI – b1000

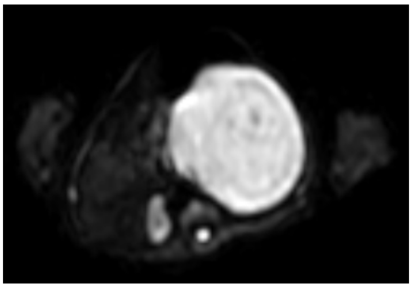

ADC-map

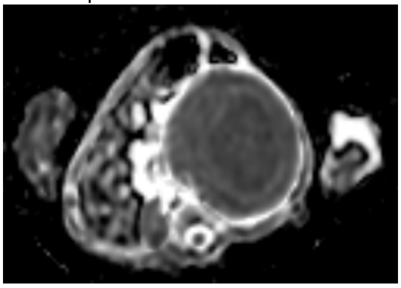

Chen et al. *Journal of Pediatric Urology*
[Specific computed tomography imaging characteristics of congenital mesoblastic nephroma and correlation with ultrasound and pathology - ScienceDirect]

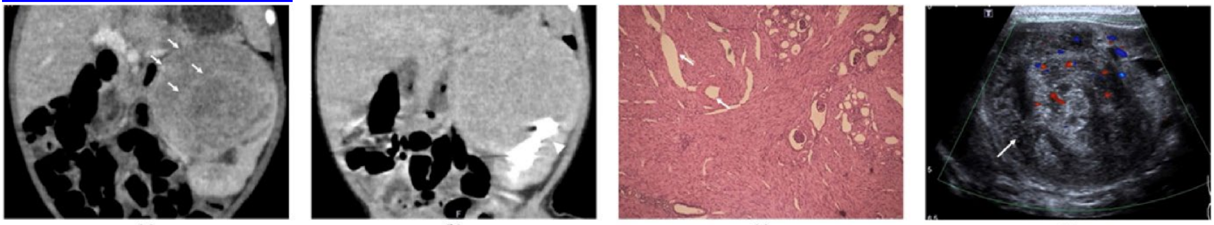

(a) (b) (c) (d)

<u>Enhancement</u>

Enhancement pattern: Indicate the predominant enhancement pattern of the tumor, following the examples below.

Homogeneous enhancement

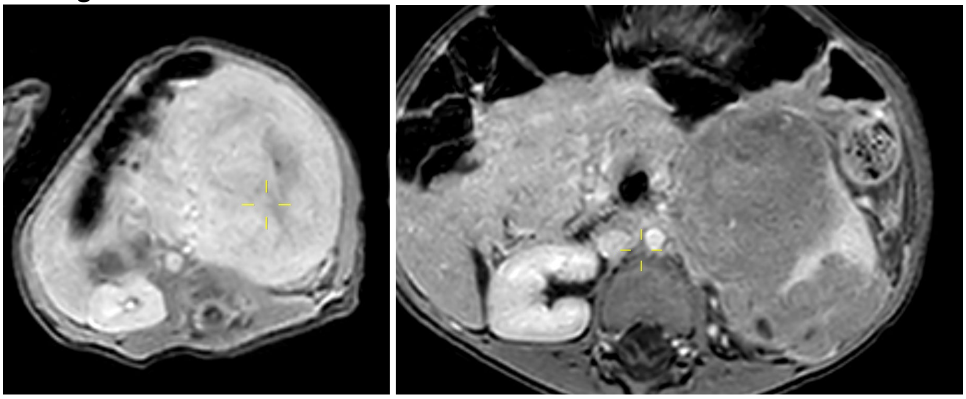

Heterogeneous enhancement

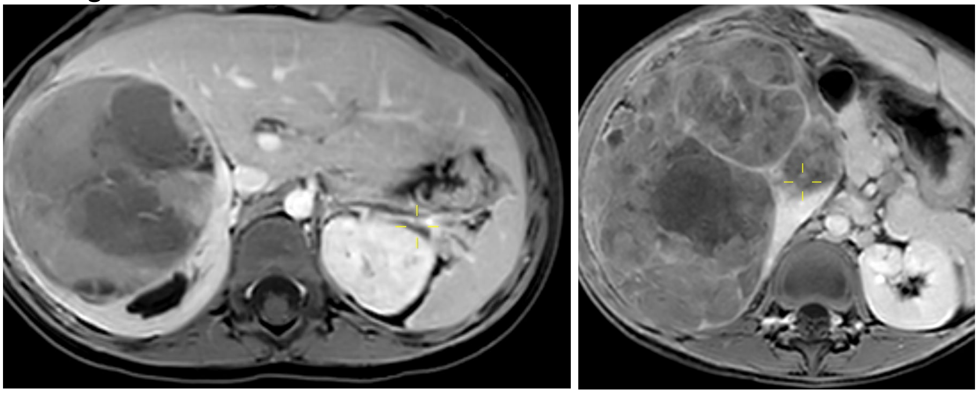

Band-like areas of late or non-enhancement

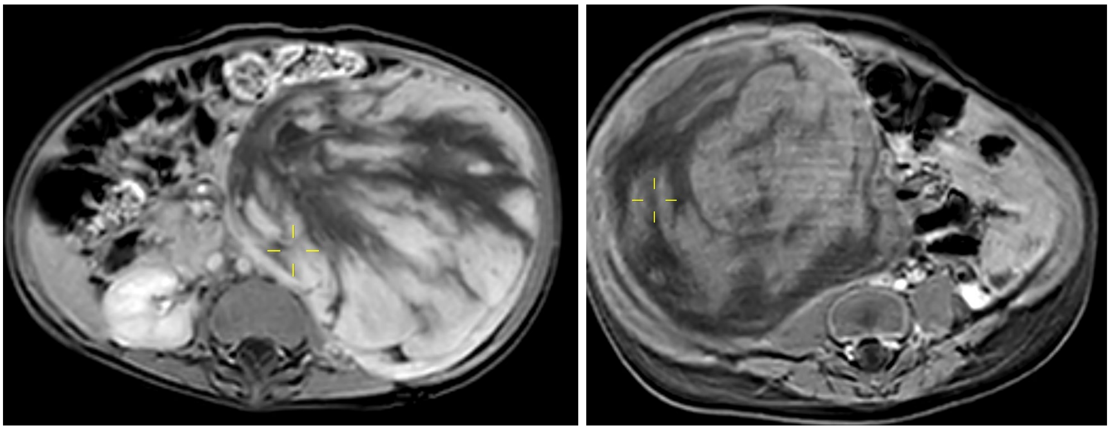

In ‘other remarks concerning the characteristics and appearance of the lesion(s)’, marked imaging characteristics and important details concerning the appearances in certain sequences can be explained and mentioned.

CRF page 4

Diffusion restriction of the lesion is defined as 'greater restriction than one would expect for this tissue', compared to the healthy (contralateral) renal tissue. Indicate the ***b-values*** used to calculate the ADC values *(for example b100/b1000)*.
Preferably two to four ***round-shaped ROIs*** (most representative part for the tumor) are drawn. These ROIs should contain only enhancing areas of the tumor (that may be visually estimated). Preference is given to four representative ROIs, but less ROIs can be reported because of limited possibilities due to tumor size, hemorrhage/necrosis, etc. Indicate the ***diameter and/or surface*** of the ROI, and indicate the (mean) ***ADC-value***. If this value is not in *10^{-3} mm^2/s, please specify the degree of magnitude of the measurement. If your ADC-measurement system gives a median ***ADC-value***, or another value, please indicate so on the CRF.
Below you can find examples of ROIs on the ADC-map, accompanied by the correlating T1W post-contrast map.

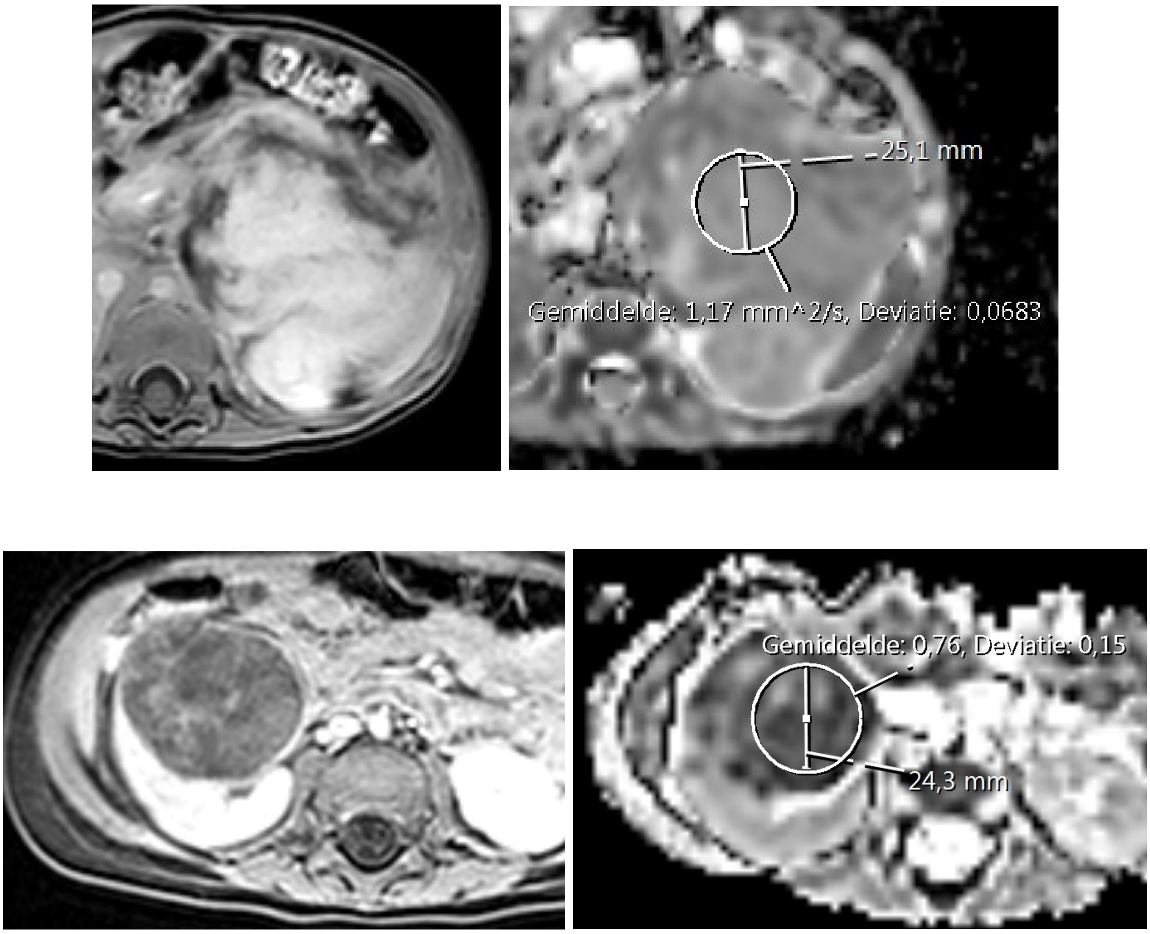

('Gemiddelde' = average/mean; 'Deviatie' = deviation)
